# Cellular Assays for Dynamic Quantification of Deubiquitinase Activity and Inhibition

**DOI:** 10.1016/j.jmb.2023.168316

**Published:** 2023-10-17

**Authors:** Seyed Arad Moghadasi, Sofia N. Moraes, Reuben S. Harris

**Affiliations:** 1 - Department of Biochemistry, Molecular Biology, and Biophysics, University of Minnesota – Twin Cities, Minneapolis, MN 55455, USA; 2 - Department of Biochemistry and Structural Biology, University of Texas Health San Antonio, San Antonio, TX 78229, USA; 3 - Howard Hughes Medical Institute, University of Texas Health San Antonio, San Antonio, TX 78229, USA

**Keywords:** cellular deubiquitinases (DUBs) USP7 and USP28, deubiquitinase (DUB) activity, deubiquitinase (DUB) inhibition, ubiquitin (Ub) biology, viral deubiquitinases (DUBs) PL^pro^ and vOTU

## Abstract

Deubiquitinases (DUBs) are proteolytic enzymes that catalyze the removal of ubiquitin from protein substrates. The critical role of DUBs in regulating protein ubiquitination makes them attractive drug targets in oncology, neurodegenerative disease, and antiviral development. Biochemical assays for quantifying DUB activity have enabled characterization of substrate preferences and discovery of small molecule inhibitors. However, assessing the efficacy of these inhibitors in cellular contexts to support clinical drug development has been limited by a lack of tractable cell-based assays. To address this gap, we developed a two-color flow cytometry-based assay that allows for sensitive quantification of DUB activity and inhibition in living cells. The utility of this system was demonstrated by quantifying the potency of GRL0617 against the viral DUB SARS-CoV-2 PL^pro^, identifying potential GRL0617 resistance mutations, and performing structure–function analysis of the vOTU domain from the recently emerged Yezo virus. In addition, the system was optimized for cellular DUBs by modifying a GFP-targeting nanobody to recruit USP7 and USP28 to benchmark a panel of reported inhibitors and assess inhibition kinetics. Together, these results demonstrate the utility of these assays for studying DUB biology in a cellular context with potential to aid in inhibitor discovery and development.

## Introduction

Ubiquitination is a dynamic post-translational modification that plays a crucial role in regulating numerous biological processes in eukaryotic organisms.^1^ This modification involves the conjugation of ubiquitin to proteins through an E1-E2-E3 enzymatic cascade that results in the formation of an isopeptide bond between the C-terminus of ubiquitin and the side chain of a lysine residue on the target protein.^2^ Ubiquitin can be added as a single molecule (monoubiquitination) or in the form of homotypic or heterotypic chains (polyubiquitination), which are conjugated iteratively to one of seven lysine residues present in ubiquitin itself.^2^ The reversal of protein ubiquitination is catalyzed by deubiquitinases (DUBs), a class of proteolytic enzymes capable of cleaving the isopeptide bond at the C-terminus of ubiquitin.^3^ Given the critical roles of protein ubiquitination in cell signaling and homeostasis, many pathogens have evolved an ability to regulate the ubiquitination status of cellular proteins to create an environment more permissive to infection.^4,5^ For instance, multiple viruses encode DUBs, which have broader substrate preferences that not only include ubiquitin but also ubiquitin-like proteins such as ISG15 and SUMO.^6,7^

As vital regulators of ubiquitin-dependent biological processes, DUBs have emerged as promising therapeutics drug targets.^8^ Initially, the conserved nature of the active site within this class of enzymes posed a challenge to the development of selective DUB inhibitors. However, recent advances in high-throughput screening campaigns, fragment-based drug discovery, and the synthesis of targeted small molecule libraries have led to the development of potent and selective inhibitors of several cellular and viral DUBs.^8,9^ A cornerstone in the characterization of DUBs and identification of inhibitors has been the development of precision chemical tools for quantifying activity.^10^ Substrates such as ubiquitin-7-amino-4-methylcoumarin (Ub-AMC) and ubiquitin-rhodamine (Ub-Rho) are widely used to measure the catalytic activity of purified DUBs, with the latter exhibiting success in high-throughput screening campaigns.^11–13^ Moreover, the synthesis of di-ubiquitin chains with different linkages has provided valuable insights into the substrate preferences of different DUBs.^3,14,15^ Comprehensive profiling of these substrate preferences has allowed DUBs to be used as tools for investigating the ubiquitination status of specific substrates and the diversity of ubiquitin chain linkages across the proteome.^16,17^

Activity based probes (ABPs) have also been valuable tools in the study of DUBs. These probes consist of ubiquitin containing an electrophile at the C-terminus that covalently labels the catalytic cysteine of active DUBs in cell lysates.^18^ By introducing an affinity handle at the N-terminus of ABPs, protein purification and subsequent mass spectrometry analysis can be performed to identify active DUBs across the proteome, which has led to the discovery and characterization of new enzymes in this class.^18–20^ The use of immunoblotting to assess the ubiquitination status of a DUB target in response to an inhibitor is a useful approach to gauge the potency of a compound. However, in cases where substrates are poorly characterized, an alternative approach that allows for semi-quantitative analysis of inhibitor potency in a cellular setting consists of pre-treating cells with an inhibitor, followed by lysis and labelling with DUB ABPs. More recently, ABPs have been coupled with advanced high-throughput mass spectrometry, enabling multiplexed assessment of inhibitor selectivity and screening to identify new covalent inhibitors across the DUBome.^9^ Although ABPs have been instrumental in the study DUBs, there is still a need for more efficient, quantitative, and high-throughput assays for measuring the activity and inhibition of DUBs in living cells.

Cellular reporter assays are valuable tools for small molecule drug discovery and mechanistic studies.^21^ By overcoming the need for protein purification and *in vitro* optimization, cellular assays enable higher throughput mutational studies to investigate protein structure–function relationships, with the additional benefit of informing about endogenous regulatory mechanisms. In the study of viral proteins as drug targets, cellular assays provide a means to quantify inhibitor efficacy without requiring high biocontainment facilities and enable the evaluation of potential resistance emergence to advanced molecules.^22–24^ In the context of the COVID-19 pandemic, there has been great interest in the papain-like protease of SARS-CoV-2 (SARS2 PL^pro^) due to its essential roles in polyprotein processing and contribution to coronavirus pathogenicity through its deubiquitinase activity.^25,26^ To date, two cellular reporter assays have been developed to quantify the peptide cleavage activity of PL^pro^ using fluorescence or luminescence as readouts, but neither can specifically quantify its DUB or deIS-Gylase activities.^27,28^

To overcome these limitations, we developed a two-color flow cytometry-based assay that specifically quantifies PL^pro^ DUB activity relative to its expression levels in cells. This assay enabled us to assess the potency of a chemical inhibitor, GRL0617, and identify PL^pro^ mutants that are less susceptible to inhibition. The potential broader utility of this system was demonstrated by characterizing the DUB activity of the viral ovarian tumor (vOTU) domain from the recently identified Yezo virus (YEZV) through a structure–function analysis. Finally, to expand assay utility to cellular DUBs, we engineered a GFP-targeted nanobody with decreased binding efficiency to mediate DUB activity-dependent stabilization of a ubiquitinated substrate and implemented it in our assay to characterize the selectivity and potency of previously reported small molecule inhibitors of cellular USP7 and USP28.

## Results

### A cellular assay for real-time DUB activity quantification

**Figure 1.**
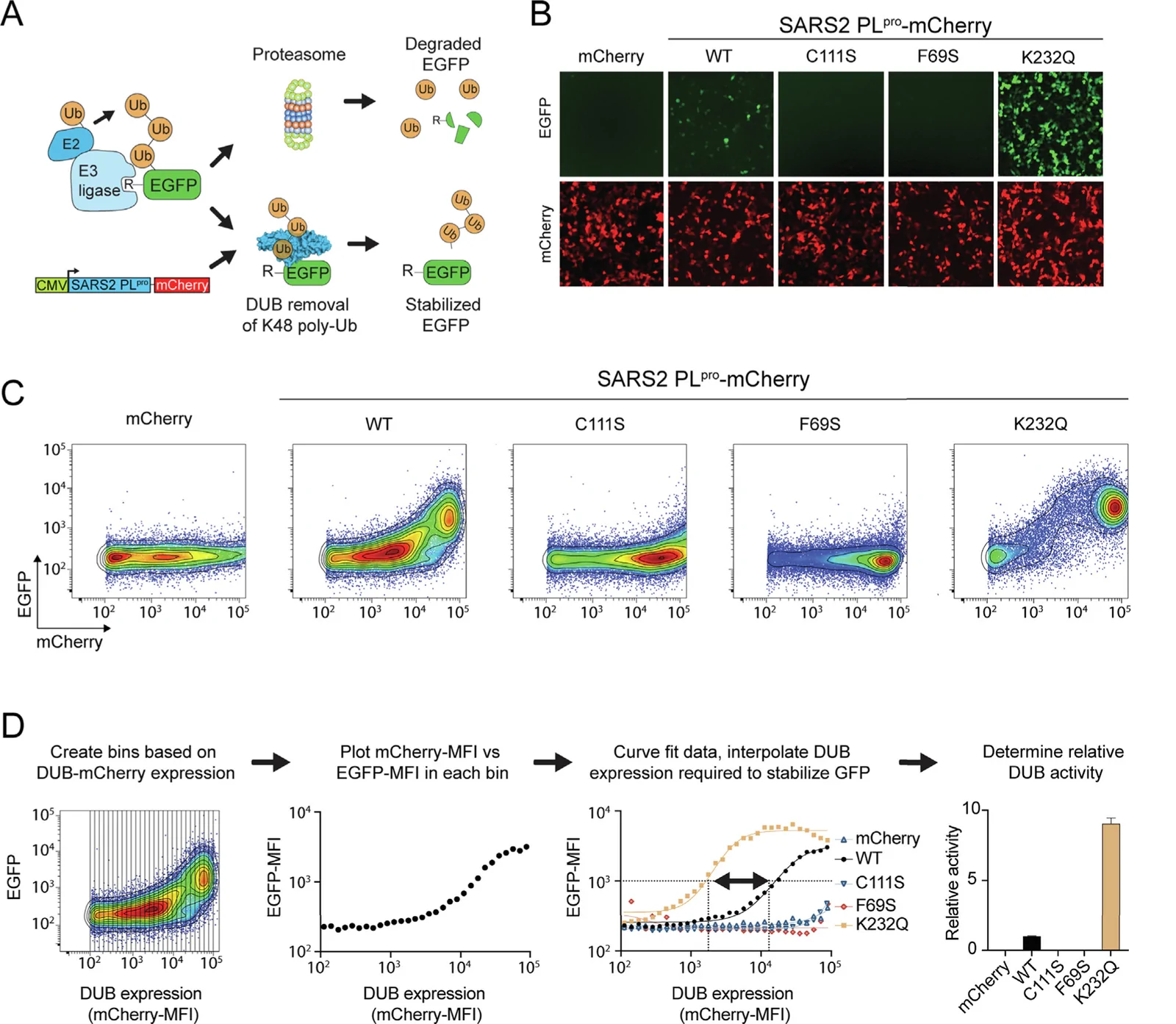
Establishing a two-color flow cytometry readout for SARS2 PL^pro^ DUB activity. (**A**) Schematic depicting a fluorescence-based DUB activity assay. Following ubiquitin fusion cleavage, the arginine residue at the N-terminus of EGFP is recognized by an UBR ligase, resulting in K48-linked poly-ubiquitination and subsequent proteasomal degradation. EGFP ubiquitination is reversed by overexpressing an active DUB such as SARS2 PL^pro^, resulting in protein stabilization and increased fluorescence. (**B**) Representative live-cell fluorescence microscopy images of 293T-Ub-R-C3 cells 24 h post-transfection with the indicated constructs. (**C**) Representative flow cytometry plots of 293T-Ub-R-C3 cells 24 h post-transfection with the indicated constructs. mCherry (X-axis) indicates DUB expression levels, GFP (Y-axis) is a proxy for DUB activity. Gating strategy is depicted in Figure S1(D). (**D**) Workflow for quantifying DUB activity relative to protein expression levels in 293T-Ub-R-C3 cells. Data in histogram are mean ± standard deviation from three independent experiments.

K48-linked ubiquitin chains serve as signals for targeting proteins for degradation by the proteasome.^29^ This property inspired the design of a DUB activity assay in which a ubiquitinated fluorescent protein is continuously degraded unless the K48-linked ubiquitin chains are removed by DUB overexpression. A reporter construct was made by employing the ubiquitin fusion technique^30^ to generate a modified version of enhanced green fluorescent protein (EGFP) with an N-terminal ubiquitin followed by an arginine (Ub-R-EGFP in Figure 1(A)). Upon post-translational cleavage of the ubiquitin fusion by cellular DUBs, the N-terminal arginine residue is recognized by UBR box-containing E3 ligases, resulting in K48-linked polyubiquitination and subsequent targeting of EGFP for proteasomal degradation.^31,32^ A clonal reporter cell line stably expressing the Ub-R-EGFP fusion was generated by retroviral transduction of 293T cells followed by screening to identify a maximally responsive clone (293T-Ub-R-C3 in Figure S1(A)). This unique cell line exhibits undetectable baseline levels of EGFP by immunoblot, fluorescence microscopy, and flow cytometry, which can be elevated over 50-fold upon proteasome inhibition (*e.g*., MG132 treatment in Figure S1(B–C)).

To test the effect of DUB overexpression on this reporter system, 293T-Ub-R-C3 cells were transfected with a plasmid encoding SARS2 PL^pro^ fused to mCherry or mCherry alone as a negative control and analyzed 24 h later by fluorescence microscopy. As expected, cells transfected with mCherry alone exhibit undetectable EGFP fluorescence (Figure 1(B–C)). In contrast, cells transfected with wildtype (WT) PL^pro^-mCherry show a clear increase in EGFP signal (Figure 1(B–C)). The DUB activity of PL^pro^ is required, as a catalytically inactive C111S mutant does not increase EGFP levels. Reporter fluorescence also remains low following transfection with a F69S mutant, which is defective in recognizing K48-linked ubiquitin chains.^33,34^ In stark contrast, a hypermorphic mutant of PL^pro^, K232Q, which exhibits higher DUB activity but normal deISGylase and peptide cleavage activities,^35^ triggers an exceptionally strong and uniform restoration of EGFP fluorescence (Figure 1(B–C)).

To improve quantification, 293T-Ub-R-C3 cells were transfected with PL^pro^ mutants fused to mCherry and analyzed 24 h later by flow cytometry (gating strategy depicted in Figure S1(D)). The intrinsic variability of each transient transfection was overcome by leveraging the observation that, within individual cells, mCherry fluorescence (DUB expression level) associates positively with EGFP fluorescence (read-out for DUB activity) (Figure 1(C)). Data from individual cells can thus be divided into logarithmic bins based on mCherry values, and dose response curves are generated by plotting the mean mCherry and EGFP fluorescence values from each bin (Figure 1(D)). The robustness of this approach is evidenced by a 9-fold improvement in the restoration of EGFP fluorescence (DUB activity) by K232Q PL^pro^ in comparison to WT PL^pro^, a value that is similar to the reported biochemical difference *in vitro* in the specific activity of these two enzymes (approximately 1 order of magnitude).^35^

### SARS2 PL^pro^ inhibitor characterization

**Figure 2.**
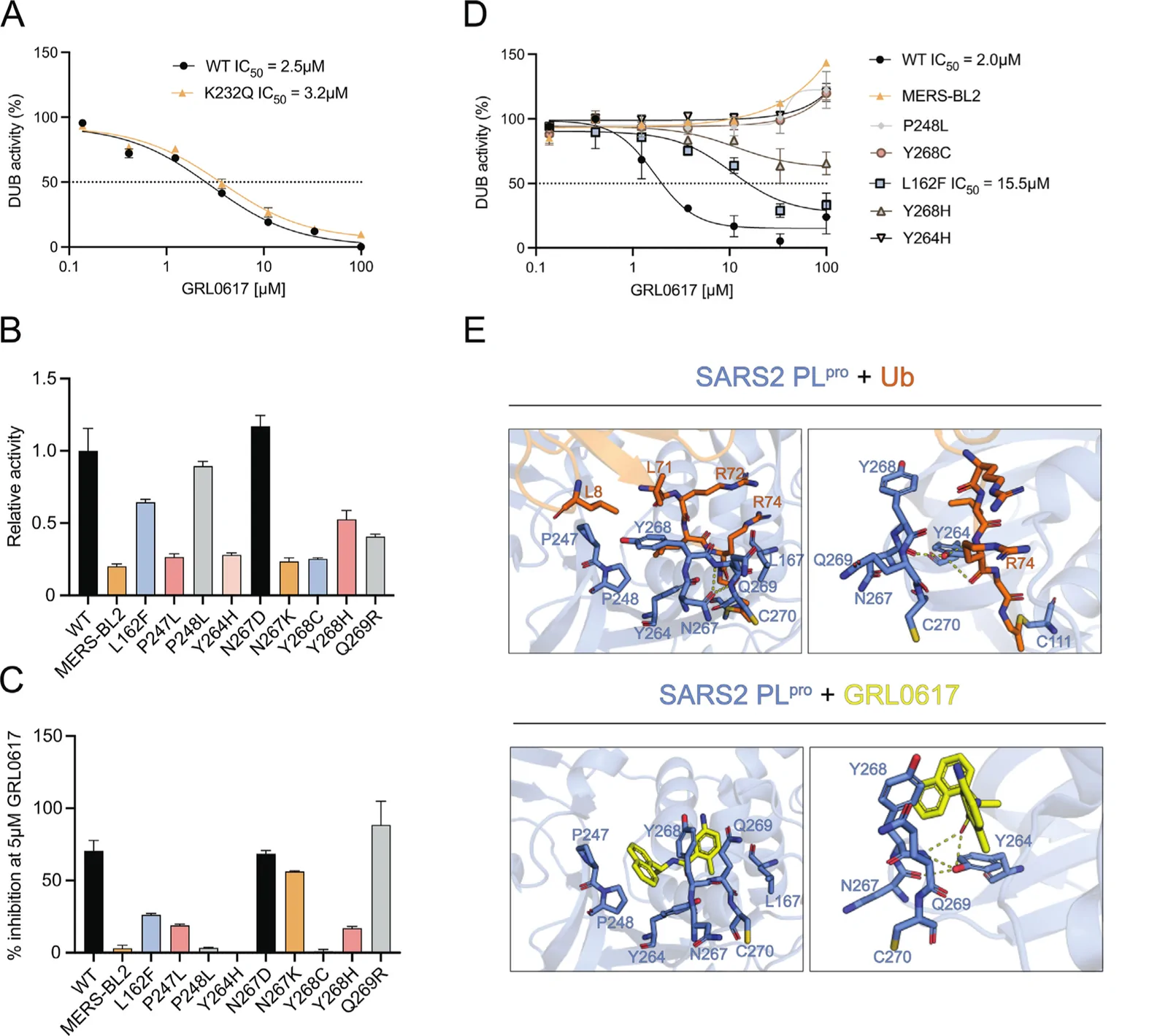
Quantification of GRL0617 cellular potency against SARS2 PL^pro^ mutants. (**A**) Dose-response curves derived from flow cytometry analysis of 293T-Ub-R-C3 cells transfected with the indicated SARS2 PL^pro^ constructs and treated GRL0617 following a 7-point 3-fold serial dilution starting at 100 mM. Data in histogram are mean ± standard deviation from three independent experiments. (**B**) Relative DUB activity of indicated SARS2 PL^pro^ mutants. The activity of the WT enzyme has been normalized to 1. Data are mean ± standard deviation from three independent experiments. (**C**) Percent inhibition of DUB activity of the indicated SARS2 PL^pro^ mutants treated with 5 μM GRL0617. Percent inhibition is relative to the catalytic activity of the respective mutant as calculated in panel B. Data are mean ± standard deviation from three independent experiments. (**D**) Dose-response curves of GRL0617 against select SARS2 PL^pro^ mutants (data are mean +/− SD of biologically independent duplicate experiments). (**E**) Crystal structures of SARS PL^pro^ (blue) in complex with ubiquitin (orange; PDB: 6XAA) and GRL0617 (yellow; PDB: 7CJM).

SARS2 PL^pro^ has received renewed attention as an antiviral drug target due to its essential proteolytic role in viral polyprotein processing and as a deubiquitinase that antagonizes host innate immune responses.^25,26^ GRL0617 is a PL^pro^ inhibitor derived from a high-throughput screen hit.^36^ To test whether our assay can be used to determine the inhibitory capacity of small molecules, 293T-UbR-C3 cells were transfected with WT and K232Q PL^pro^, treated 6 h post-transfection with increasing concentrations of GRL0617, and analyzed 18 h later by flow cytometry. GRL0617 causes a clear dose responsive decrease in accumulation of EGFP fluorescence for both constructs with IC_50_ values of 2.5 and 3.2 μM, respectively (Figure 2(A), S2(A)). These inhibitory concentrations are similar to those determined using purified enzymes and a fluorescent peptide substrate (2.1–2.3 μM).^37,38^ Time-lapse fluorescence microscopy imaging of cells transfected with SARS2-PL^pro^ and subsequently treated with 10 μM GRL0617 24 h post-transfection show a time dependent decrease in EGFP signal beginning at 2 h post-treatment (Figure S2(B)).

GRL0617 selectively inhibits SARS2 PL^pro^ and is inactive against the homologous enzymes from MERS and NL63, the basis of which has been ascribed to differences in the blocking loop 2 (BL2) region of these enzymes.^36,39^ To further investigate the impact of amino acid variations around the GRL0617 binding site, we tested a panel of structurally informed SARS2 PL^pro^ mutants using our system. First, we found that swapping the BL2 loop SARS2 PL^pro^ with that of MERS (resulting chimeric construct referred to as PL^pro^-BL2) causes an approximately 5-fold decrease in DUB activity (Figure 2(B)). This result is consistent with a previous report showing that this same mutant has decreased activity against a K48 linked tri-Ub substrate in a gel-based assay.^40^ However, despite lower DUB activity, PL^pro^-BL2 shows almost no sensitivity to 5 μM GRL-0617 (<2% inhibition; Figure 2(C)), further underscoring the consistency between our cell-based system and reported biochemical data.

Several single amino acid substitution mutants also exhibit decreased DUB activity (P247L, Y264H, N267K, Y268C, Y268H, and Q269R), but a few maintain near WT DUB activity (L162F, P248L, and N267D; Figure 2(B)). Among the mutant panel, N267D, N267K, and Q269R have little impact on GRL0617 potency, L162F, P247L cause a slight decrease in potency (2- to 5-fold), and P248L, Y264H, Y268C and Y268H show strong decreases in potency (>10-fold; Figure 2(C)). Dose response curves demonstrate that P248L, Y264H, and Y268C cause near-complete resistance, Y268H shows some inhibition at high inhibitor concentrations but no more than 50%, and L162F exhibits an intermediate resistance phenotype (near 8-fold increase in IC_50_; Figure 2(D), S2(C)).

Y268 makes hydrophobic contacts with GRL0617 and the side chains of L71 and L73 of ubiquitin. Therefore, changing this residue to Cys or His is predicted to disrupt these hydrophobic interactions and account for decreased DUB activity and GRL0617 sensitivity (Figure 2(E)). GRL0617 resistance and lower DUB activity of Y264H may be due to the loss of a network of inter- and intra-molecular hydrogen bonds between the hydroxyl group of Y264, GRL0617, and the backbone amide of ubiquitin R74. The complete loss of GRL0617 sensitivity by the P248L mutation is not as clear structurally. Due to the proximity of P248 and the β-hairpin containing the BL2 loop, mutation to a bulkier Leu sidechain may disrupt the structural conformation adopted by the substrate binding pocket and occlude GRL0617 binding. Indirect disruption of the BL2 loop may also be at play in the slight resistance observed with the L162F mutation. Disruption of the intramolecular hydrogen bonds between the side chain carbonyl of N267 to the backbone amines of Q269 and C270 may explain the differential impact of N267K and N267D on the DUB activity of SARS2 PL^pro^. Taken together, these data demonstrate the ability of our assay to specifically quantify the impact of variation on DUB activity and GRL0617 inhibition of SARS2 PL^pro^.

### Extension to additional viral DUBs

**Figure 3.**
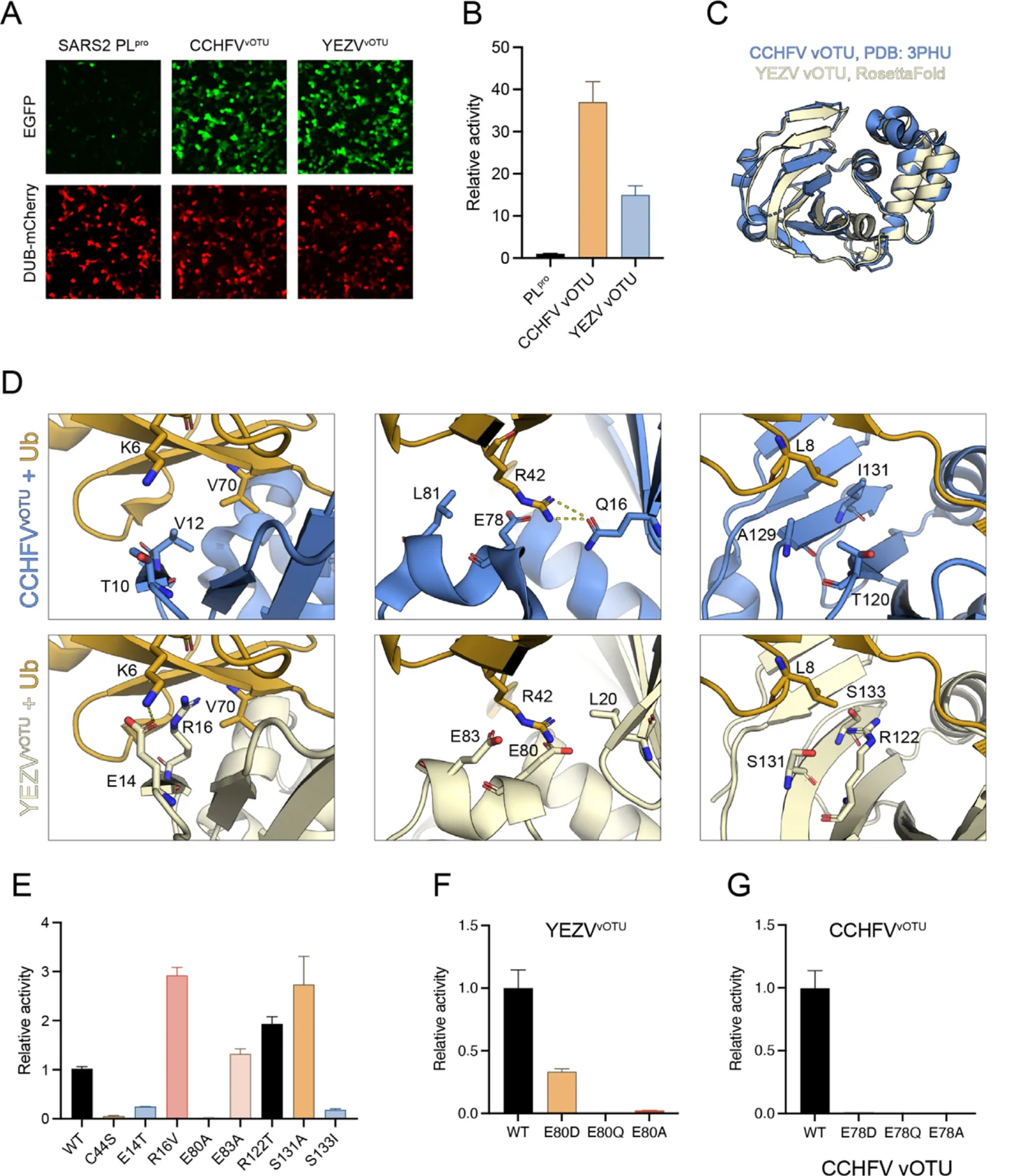
Characterization of the DUB activity of YEZV vOTU domain. (**A**) Representative live-cell fluorescence microscopy images of 293T-Ub-R-C3 cells 24 h post-transfection with the indicated constructs. (**B**) Relative DUB activity of CCHFV^vOTU^ and YEZV^vOTU^. The activity SARS2 PL^pro^ has been normalized to 1. Data are mean ± standard deviation from three independent experiments. (**C**) Crystal structure of CCHFV vOTU (blue; PDB: 3PHU) overlayed with a structural model of YEZV vOTU predicted using RosettaFold (off-white). (**D**) Top: zoom-in of the binding interface of CCHFV vOTU (blue) and ubiquitin (gold) (PDB: 3PHW). Bottom: putative binding interface of YEZV vOTU (off-white; predicted) and ubiquitin (gold) based on a structural overlay with CCHFV vOTU (PDB: 3PHU). (**E**) Relative DUB activity of the indicated YEZV vOTU mutants. The activity of WT YEZV vOTU has been normalized to 1. Data are mean ± standard deviation from two independent experiments. (**F**) Relative DUB activity of the indicated YEZV vOTU mutants. The activity of WT YEZV vOTU has been normalized to 1. Data are mean ± standard deviation from two independent experiments. (**G**) Relative DUB activity of the indicated CCHFV vOTU mutants. The activity of WT CCHFV vOTU has been normalized to 1. Data are mean ± standard deviation from two independent experiments.

We next sought to demonstrate the broader utility of our 293T-Ub-R-C3 system by testing other viral DUBs. Nairoviruses encode a DUB domain at the N-terminal end of the viral L-protein that belongs to the ovarian tumor protease (OTU) family.^41^ This viral OTU (vOTU) domain plays a key role in innate immune evasion through deconjugation of ubiquitinated and ISGylated substrates.^42^ The vOTU domain of Crimean Congo Hemorrhagic Fever Virus (CCHFV) is the most well-studied within this viral family and exhibits high DUB activity with the capacity to cleave K48 ubiquitin linkages.^42,43^ A new nairovirus associated with febrile illness, Yezo Virus (YEZV), was discovered recently in Hokkaido, Japan.^44^ The catalytic activity of the YEZV vOTU domain has yet to be characterized. Therefore, the vOTU domains of CCHFV and YEZV were cloned into expression plasmids with a C-terminal mCherry tag and transfected into 293T-UbR-C3 cells to compare their DUB activities to SARS2 PL^pro^. The CCHFV^vOTU^ exhibits approximately 40-fold higher DUB activity compared to SARS2 PL^pro^ (Figure 3(A–B)), consistent with published *in vitro* kinetic data.^34,43,45,46^

The YEZV^vOTU^ also shows approximately 15-fold higher activity relative to SARS2 PL^pro^ (Figure 3(A–B)). The strong DUB activity of the YEZV^vOTU^ was surprising given low sequence similarity (<10%) to the well-characterized CCHFV^vOTU^ (Figure S3(A–B)). To investigate the structural determinants of YEZV^vOTU^ activity, RoseTTAFold^47^ was used to generate an atomic model. Despite low primary sequence similarity, the predicted structure of the YEZV^vOTU^ superimposed well with the CCHFV^vOTU^ crystal structure (RMSD = 1.012 angstroms) (Figure 3(C)).

We next overlayed this model onto the co-crystal structure of Ub-bound CCHFV^vOTU^ to identify residues that impact DUB activity (Figure 3(D)). Three regions were predicted to be at the interface of YEZV^vOTU^ and ubiquitin. The N-terminus contains an antiparallel beta sheet (1–2) unique to vOTUs, which has been shown to be essential for DUB activity.^43^ To assess the importance of this viral protein motif, two amino acid substitutions were constructed, E14T and R16V, and shown to have opposite effects (Figure 3(E)). E14T causes a decrease in DUB activity in YEZV^vOTU^, which could be rationalized by the proximity of E14 to K6 of ubiquitin, consistent with disruption of an electrostatic interaction (Figure 3(D)). In contrast, R16V increases YEZV^vOTU^ activity, which may be due to increased hydrophobic contacts with V70 of ubiquitin, analogous to what is observed in the ubiquitin bound CCHFV^vOTU^ structure (Figure 3(D)).

An important role was shown for hydrophobic interactions between the vOTU domains and L8 of ubiquitin.^48^ However, because this region is dominated by hydrophilic amino acids in YEZV^vOTU^ (Figure 3(D)), we mutated R122 and S131 in YEZV^vOTU^ to their more hydrophobic counterparts in CCHFV^vOTU^ to test whether this might increase activity (Figure S3(A)). As predicted, R122T and S131A causes an increase in DUB activity, whereas S133I causes an unexpected reduction. These results combine to suggest that YEZV^vOTU^ may engage ubiquitin in a distinct orientation that is unable to accommodate an Ile at this position (Figure 3(E)).

The interaction between Q16 of CCHFV^vOTU^ and R42 of ubiquitin has been shown to be critical to its DUB activity, however YEZV^vOTU^ has a Leu at the analogous position (L20, Figure 3(D), S3A).^43^ Instead, in proximity lies E80, which is conserved in CCHFV^vOTU^ (E78), and E83 further down the helix, which is not conserved. The proximity of these two residues suggests a contribution to the electronegativity of this pocket which could accommodate the positive charge of ubiquitin R42. Interestingly, E80A causes a 50-fold decrease in DUB activity, while E83A shows activity identical to that of the WT protein (Figure 3(E)). More conservative amino acid changes at this position both in YEZV^vOTU^ and CCHFV^vOTU^ were tested to determine if they still retained activity. For CCHFV^vOTU^, E78D, E78Q, and E78A all lead to >100-fold decreases in DUB activity, indicating that this glutamic acid residue is critical (Figure 3(F)). YEZV^vOTU^ behaves slightly differently, with E80Q abolishing activity while E80D only causes approximately 3-fold less activity than WT (Figure 3(F)). Despite predicted three-dimensional structural conservation, these results suggest that the very divergent sequence space that YEZV^vOTU^ occupies has led to an altered ubiquitin binding mode, which may explain some of the observed differences relative to CCHFV^vOTU^. Nevertheless, these results combine to demonstrate the utility of the assay to interrogate both existing and predicted viral DUBs.

### Assay modification for cellular DUBs

**Figure 4.**
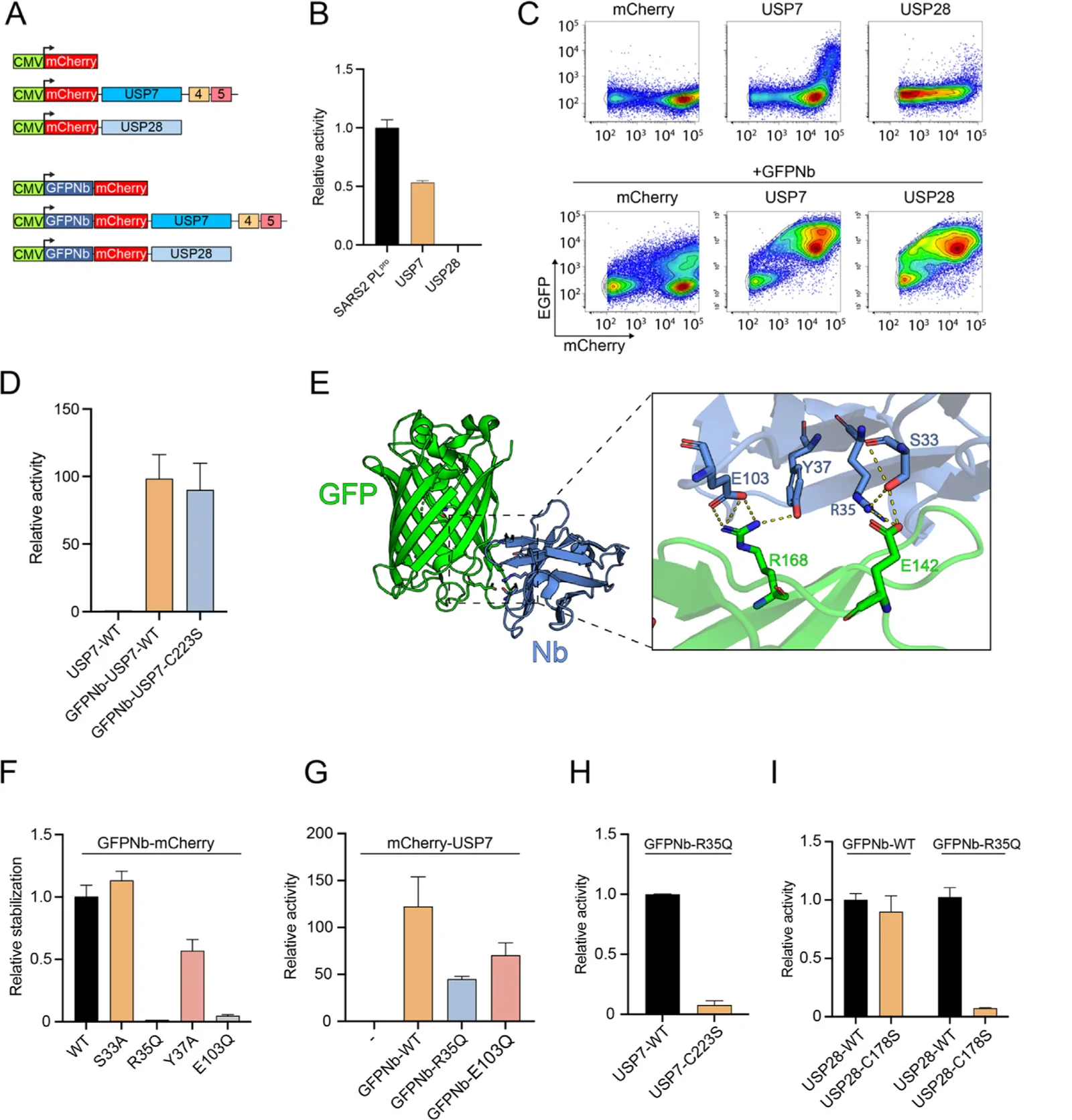
Structurally informed mutagenesis of a GFP nanobody enables DUB-dependent EGFP stabilization. (**A**) Schematic of constructs for USP7, USP28 and mCherry control chimeric plasmids used in panels B-C. (**B**) Relative DUB activity of USP7 and USP28 compared to SARS2 PL^pro^. The activity of SARS2 PL^pro^ has been normalized to 1. Data are mean ± standard deviation from two independent experiments. (**C**) Representative flow cytometry plots of 293T-Ub-R-C3 cells 24 h post-transfection with mCherry, mCherry-USP7, and mCherry-USP28 (top) or the same constructs fused to a GFP-targeting nanobody (GFPNb) (bottom). (**D**) Activity of WT and catalytically inactive GFPNb-mCherry-USP7 fusion constructs relative to mCherry-USP7, which is normalized to 1. (**E**) Crystal structure of the vHHGFP4 nanobody (blue) bound to GFP (green) (PDB: 3OGO). Zoom-in highlights the nanobody residues selected for mutational analysis. (**F**) Relative stabilization of EGFP in 293T-Ub-R-C3 cells transfected with the indicated GFPNb-mCherry fusion constructs. The stabilization in cells transfected with mCherry fused to the WT nanobody has been normalized to 1. Data are mean ± standard deviation from two independent experiments. (**G**) Relative DUB activity of USP7 in 293T-Ub-R-C3 cells transfected with mCherry-USP7 (−) or the indicated GFPNb-mCherry-USP7 fusions. The activity of mCherry-USP7 has been normalized to 1. Data are mean ± standard deviation from three independent experiments. (**H**) Relative dependence on the catalytic activity of the GFPNb^R35Q^-USP7 fusion construct for EGFP stabilization in 293T-Ubr-R-C3 cells. The activity of the catalytically active (WT) USP7 construct has been normalized to 1. Data are mean ± standard deviation from two independent experiments. (**I**) Relative dependence on the catalytic activity of GFPNb^WT^-USP28 and GFPNb^R35Q^-USP28 fusion constructs for EGFP stabilization in 293T-Ubr-R-C3 cells. The activity of the corresponding catalytically active (WT) constructs has been normalized to 1. Data are mean ± standard deviation from two independent experiments.

Cellular DUBs are also emerging as drug targets due to critical contributions to the ubiquitination status of proteins implicated in multiple diseases.^49^ Thus, the ability to quantify the enzymatic activity of host-encoded DUBs in a cellular setting could become an asset in understanding the biology of these enzymes in their natural environment, as well as for the validation of small molecules in the drug discovery process. To test the capacity of our assay to quantify the activity of cellular DUBs, we focused on USP7 and USP28, which are well-studied enzymes with existing small molecule inhibitors.^12,50–53^ A USP7 expression plasmid was generated encoding mCherry at the N-terminus followed by the catalytic DUB domain, a flexible linker, and the Ubl4–5 domains (schematic in Figure 4(A)). This domain organization is reported to exhibit catalytic activity similar to the full-length enzyme.^54,55^ A USP28 construct was generated similarly with mCherry fused to the N-terminus of the catalytic domain and separated by a flexible linker (schematic in Figure 4(A)). However, transfection into the 293T-UbR-C3 reporter cell line yielded modest EGFP fluorescence for the USP7 construct and no detectable fluorescence for USP28 (Figure 4(B)). Furthermore, the relative activity of USP7 was less than WT SARS2 PL^pro^ (Figure 4(B)), which was unexpected given its reported catalytic efficiency in biochemical assays with fluorescent ubiquitin substrates.^34,45,54–56^ The weaker activity of these cellular DUBs may be due to regulatory mechanisms that likely serve to control the activity of endogenous DUBs and prohibit unhinged deubiquitination. Alternatively, longer K48 poly-Ub chains may be suboptimal substrates for these enzymes (*e.g*.^57^). Furthermore, apo structures of USP7 and USP28 contain misaligned catalytic triads, unlike PL^pro^, which could indicate that the basally inactive conformations of these DUBs enable activity only when recruited to their intended substrates in a cellular setting.^54,56,58–60^

Nanobodies have been shown to facilitate the recruitment of DUBs to target proteins.^61^ We therefore tested whether fusion of an anti-GFP nanobody (GFPNb^62^) to these cellular DUBs might help improve reporter signal (schematic in Figure 4(A)). As anticipated, fusion to the N-terminus of the mCherry-tagged USP7 construct leads to a strong increase in reporter stabilization, as shown by increased GFP signal following transfection into 293T-UbR-C3 cells (Figure 4(C), middle FACS profiles). However, fusion of the nanobody to mCherry alone also leads to reporter stabilization, albeit less than that attributable to the fused DUB (Figure 4(C), left FACS profiles). Of greater concern, mutation of the catalytic cysteine of USP7 in the nanobody fusion construct also leads to stabilization levels nearly indistinguishable from the active counterpart, which undermines the utility of this initial construct for quantification of deubiquitination activity (Figure 4(D)).

The stabilization of Ub-R-GFP by Nb-mCherry (without a DUB fusion) could be a consequence of a high nanobody binding affinity to EGFP, which may sterically shield ubiquitination sites and/or limit the rate of EGFP unfolding, which is a prerequisite to degradation by the proteasome. Furthermore, inactive DUB fusions stabilize EGFP more than mCherry-Nb alone, which could be due to stable occupancy and multivalent binding of the substrate, with the catalytic domain binding to a ubiquitinated form of EGFP, further limiting access to the proteasome and/or blocking poly-Ub chain elongation. Regardless of the exact mechanism, we reasoned that these issues might be overcome by introducing rationally designed amino acid substitutions to decrease the binding efficiency of the nanobody. Using an available crystal structure of the GFP-nanobody complex,^63^ several mutants were designed to weaken but not completely disrupt the interaction (Figure 4(E)). Although Nb S33A and Y37A only lead to minimal diminution of reporter stabilization, R35Q and E103Q cause 50- and 100-fold decreases in stabilization, respectively (Figure 4(F)). These unique R35Q and E103Q mutations when introduced into the Nb-mCherry-USP7 fusion construct still retain high levels of EGFP stabilization, albeit 2- and 3-fold less than the WT Nb fusion, respectively (Figure 4(G)). When combined, the R35Q/E103Q double mutant abolishes the nanobody dependent increase in USP7 activity, highlighting the critical nature of the interactions at the GFP-nanobody interface for the observed phenotype (Figure S4). Most importantly, the R35Q mutant Nb fusion now exhibits a clear dependence on the catalytic activity of USP7 (Figure 4(H)). A similar catalytic dependence is also evident for Nb(R35Q)-USP28 fusions, indicating that this strong reporter stabilization phenotype is likely to be generalizable to the catalytic domains of other cellular DUBs (Figure 4(I)).

### Analysis of cellular DUB inhibitors

**Figure 5.**
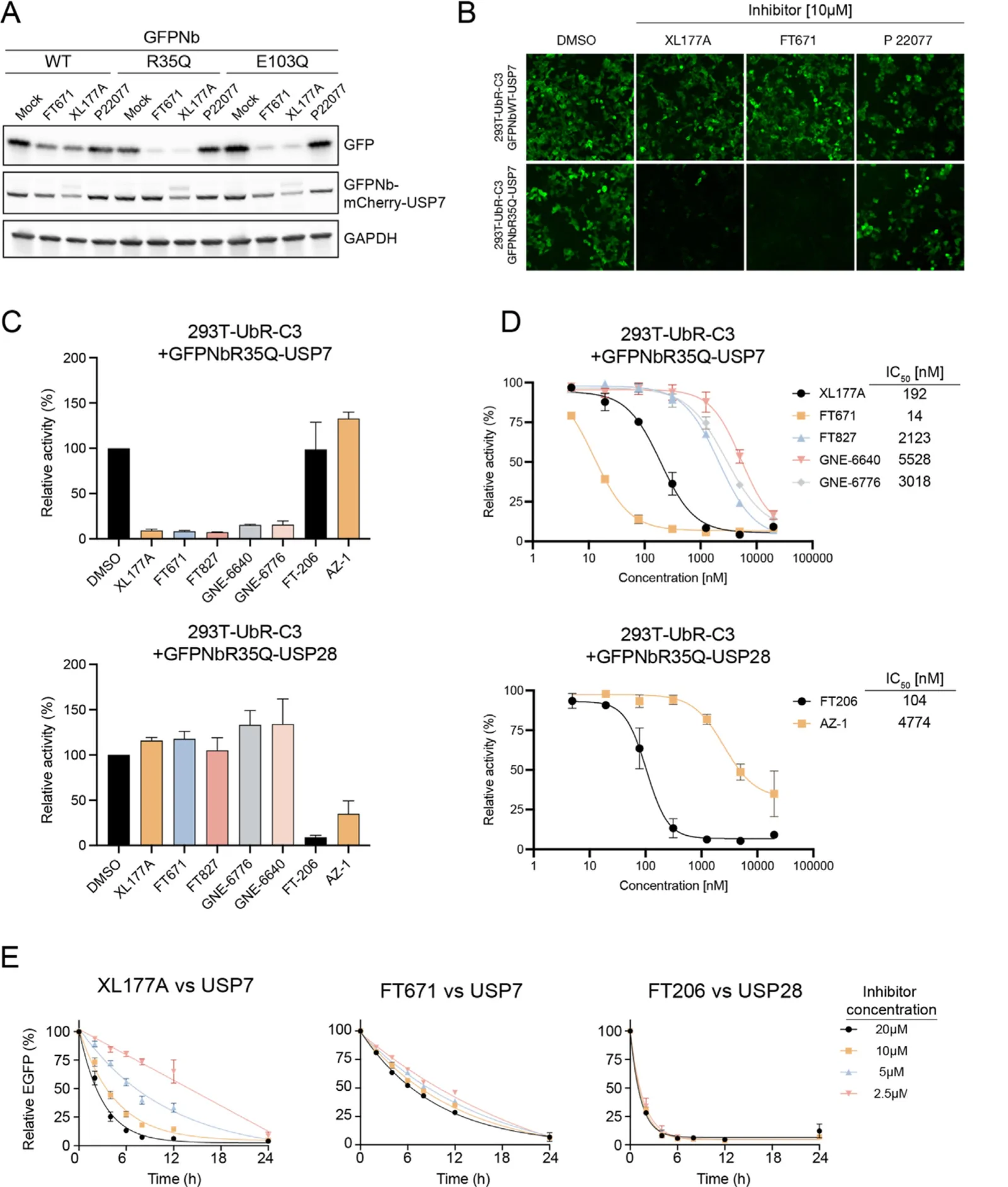
Live cell quantification of inhibitor potency and selectivity against USP7 and USP28. (**A**) Representative immunoblot of 293T-Ub-R-C3 cells stably expressing the indicated GFPNb-USP7 fusions and treated for 24 h with 10 μM of the indicated USP7 inhibitors. (**B**) Representative live cell fluorescent microscopy images of 293T-Ub-R-C3 cells stably expressing GFPNb^WT^-USP7 (top) or GFPNb^R35Q^-USP7 (bottom) and treated for 24 h with 10 μM of the indicated USP7 inhibitors. Expanded panel of cell lines and matched mCherry controls in Figure S3(C). (**C**) Relative DUB activity in 293T-Ub-R-C3 cells stably expressing GFPNb^R35Q^-USP7 (top) or GFPNb^R35Q^-USP28 (bottom) after treatment with 20 μM of the indicated small molecules for 24 h. Data are mean ± standard deviation from two independent experiments. (**D**) Dose-response curves derived from flow cytometry analysis of 293T-Ub-R-C3 cells stably expressing GFPNb^R35Q^-USP7 (top) or GFPNb^R35Q^-USP28 (bottom) after 24 h of treatment with the indicated selective inhibitors following 7-point 4-fold serial dilution beginning at 20 mM. Data are mean ± standard deviation from two independent experiments. (**E**) Kinetics of EGFP degradation in 293T-Ub-R-C3 cells stably expressing GFPNb^R35Q^-USP7 (left and center) or GFPNb^R35Q^-USP28 (right) after treatment with the indicated inhibitors for 2, 4, 6, 8, 12 and 24 h at the indicated concentrations.

We next generated cell pools stably expressing WT, R35Q, and E103Q Nb-mCherry-USP7 fusions by retroviral transduction to enable quantification of inhibitor potency without the need for repeated transfection (Figure S5(A)). In the absence of inhibitors, all three cell lines show substantial EGFP stabilization that correlates with Nb-mCherry-USP7 expression levels (Figure S5(B)). To validate the responsiveness of the cell lines to inhibitors, we used USP7 inhibitors FT671, XL177A, and P22077.^12,51,64^ Cells were treated with 10 μM compound for 16 h and then harvested for immunoblotting and fluorescent microscopy to determine EGFP levels. Consistent with the observation above of catalytic independent stabilization of EGFP when USP7 is fused to the WT GFP nanobody, only a weak (if any) decrease in fluorescence levels is observed with inhibitor treatment (Figures 5(A–B), S5(C)). This further suggests that the affinity of this interaction is too high to be disrupted, even by very potent inhibitors such as FT671 and XL177A. In contrast, cells stably expressing the R35Q or E103Q nanobody fusions show a substantial decrease in EGFP levels when treated with XL177A and FT671, consistent with USP7 DUB activity inhibition (Figures 5(A–B), S5(C)). However, P22077 shows no inhibitory activity regardless of detection method (Figures 5(A–B), S5(C)).

Given that fusion with the R35Q-Nb showed slightly greater responsiveness to chemical inhibition than the E103Q counterpart, we proceeded with the former chimeric design and generated a stable USP28 reporter cell line in a similar manner. With both established cell lines, we expanded our panel of inhibitors to include additional reported inhibitors of USP7 (FT827, GNE6640, and GNE6776) and USP28 (FT206 and AZ1).^12,50,52,53^ To test the selectivity of the compounds against these DUBs in a cellular environment using our assay, both cell lines were treated with 20 μM inhibitor for 16 h and then inhibition was quantified by flow cytometry. As expected, all USP7 inhibitors cause >50% reduction in EGFP levels without showing any inhibition in the USP28 cell line (Figure 5(C), top vs bottom bar graph). FT206 and AZ1 show the opposite effect, with no inhibition of USP7 and potent inhibition of USP28 (Figure 5(C), bottom vs top bar graph). To further quantify the potency of these compounds against their respective targets, a 7-point dose response was performed to determine cellular IC_50_ values (Figure 5(D)). FT671 and XL177A show very high potency against USP7 with IC_50_ values of 14 nM and 192 nM, respectively. The potency of FT671 in our assay was concordant with its IC_50_ of 52 nM in a ubiquitin-rhodamine assay and its ability to block proliferation of MM.1S cells with an IC_50_ of 33 nM^12^ and, importantly, demonstrated the sensitivity of this readout in the low nM concentration range. XL177A has a reported IC_50_ of 0.34 nM against purified USP7 and Ub-AMC substrate, which is much lower than what is observed in our live cell assay. However, this may be due to the much larger size of XL177A compared to FT671, which could affect permeability among other possible parameters. FT827, GNE6640, and GNE6776 all show potency in the low micromolar range, again in line with previous studies.^12,50^ FT206 also shows potent inhibition in a cellular context of approximately 104 nM, consistent with its capacity to inhibit proliferation of KF LSCC cells.^52^ AZ1 was comparatively much weaker in its capacity to inhibit USP28 in a cellular context, with approximately 50% inhibition at 20 μM (higher concentrations caused cytotoxicity and were not evaluated in-depth).

We next tested whether EGFP degradation rates might enable estimates of the kinetics of enzyme inhibition. Cells were treated with increasing concentrations of the most potent inhibitors (FT671 and XL177A for USP7 and FT206 for USP28) and harvested for flow cytometry at multiple time points over 24 h to quantify relative EGFP levels. To our surprise, although FT671 shows superior potency (lower IC_50_), inhibition of USP7, XL177A induces much faster rates of degradation of the EGFP reporter compared to FT671 at high drug concentrations (10 and 20 mM, Figure 5(E)). In contrast, at lower concentrations (2.5 mM) the opposite is observed, with XL177A inducing substantially slower degradation rates. Although many explanations for these differences are plausible, these results may suggest that the potential pharmacokinetic barriers to XL177A cellular potency can be overcome at high concentrations and enable a more rapid engagement of USP7 compared to FT671, consistent with its superior *in vitro* potency. FT206 inhibition of USP28 induces the fastest degradation of the EGFP reporter, indicating rapid target engagement, but this compound shows little dose-dependence at the concentrations tested. Different DUB substrates will likely exhibit distinct degradation rates (assuming a K48-linked ubiquitin chain architecture) upon inhibition, which are likely influenced by rates of transcription/translation and substrate ubiquitination, however our cell-based assay enables head-to-head comparison of the kinetics of inhibitor target engagement. Taken together, these results establish the utility of this Nb-modified assay system for sensitive quantification of the kinetics of cellular DUB inhibition potency and efficiency in living cells.

## Discussion

Major ongoing efforts seek a deeper understanding of DUB biology and the contributions of these enzymes to different diseases, with recently developed chemical probes serving as critical tools.^8,65^ Here, we have developed an assay for assessing the catalytic activity of both viral and host-encoded DUBs within a cellular context. First, we established a two-color flow cytometry approach to measure the stabilization of a polyubiquitinated substrate by SARS2 PL^pro^ relative to its expression levels. Using this system, we performed a mutational analysis to identify SARS2 PL^pro^ residues involved in GRL-0617 binding and described how mutating these residues can impact DUB activity. Next, we extended the assay to characterize the activity of the recently discovered YEZV vOTU in comparison to the previously described CCHFV vOTU.^43,46,48^ Finally, we adapted the assay to study host-encoded DUBs by introducing structurally informed mutations in an anti-GFP nanobody to decrease its binding capacity and enable the assessment of potency and selectivity of small molecule inhibitors against USP7 and USP28. Using engineered cell lines, we performed enzyme kinetic studies in live cells which revealed significant differences in target engagement rates among a panel of small molecule inhibitors. Taken together, our results have demonstrated the utility these cellular assays as tools for studying DUB biology and their potential to complement existing biochemical methods for investigating DUB activity and inhibition.

Although the specific cellular substrates of most viral DUBs remain unclear, the activity of these enzymes has been shown to increase viral pathogenicity and facilitate innate immune evasion^25,42,66^. Similarly to other viral proteins that act as immune antagonists, viral DUBs exhibit high sequence diversity, even within the same viral family, making it challenging to infer activity and substrate preferences.^67^ The assay described here is anticipated to be a strong complement to traditional biochemical methods, enabling the quantification of DUB activity in living cells and providing insights into how genetic variations impact enzymatic activity, particularly in the context of viruses such as SARS2 where emerging variants often harbor changes in PL^pro^. Furthermore, viral DUBs are attractive targets for antiviral drug development. However, the emergence of resistance poses a threat to the effectiveness of such therapeutics. Our assay’s capability to measure inhibitor activity against mutant enzymes facilitates the study of resistance mechanisms during the drug development process, highlighting its potential to aid in the identification of novel antiviral compounds.

Significant progress has been made in developing chemical tools to study DUB biology, which has helped to accelerate the discovery of inhibitors of cellular DUBs.^8,10^ Compound validation often relies on activity-based probes added to cell lysates or immunoblotting for the Ub status of a known target protein to monitor its ubiquitination or stability as a proxy for DUB activity. Although these are valuable approaches, such methods have limitations in terms of lower throughput and quantitative range. The reporter cell lines for USP7 and USP28 described here overcome these issues and enable quantification of target engagement with high sensitivity and no need for prior knowledge of a specific DUB substrate. Moreover, this experimental system may help overcome challenges associated with the expression and purification of certain DUBs or their mutants by expressing these enzymes in their natural cellular environment. Furthermore, the high signal-to-noise ratio (approximately 20-fold difference between samples with and without inhibitor) and reproducibility of this assay highlight its potential as a screening platform for identifying novel inhibitors.

Although the assays described here are promising tools for evaluating the catalytic activity and inhibition of DUBs in a cellular context, it is important to acknowledge limitations. First, the EGFP substrate used in this assay is conjugated with K48-linked polyubiquitin chains, which makes it suitable for studying DUBs with broad substrate specificity or a preference for K48-linked chains. However, adapting the assay for DUBs with alternative Ub linkage preferences such as K63 may prove challenging. Second, although fusion to a crippled anti-GFP nanobody successfully broadened the repertoire of DUBs that can be studied using our approach, recruitment to an artificial substrate may not fully replicate the complex biology involved in recognizing endogenous substrates. In cases where the natural substrates of DUBs are known, it may be possible to adapt the two-color flow cytometry approach described here to be able to report the degradation status of the physiologically relevant target.

The fusion of USP7 and USP28 to the R35Q anti-GFP nanobody significantly improved assay performance for inhibitor testing and validation. However, the relatively low or undetectable activity observed when these DUBs are overexpressed without the nanobody suggests additional interesting avenues for future research. Previous *in vitro* enzymatic assays have shown that USP7 exhibits significantly higher activity than SARS2 PL^pro^.^34,45,54–56^ However, surprisingly, in a cellular context using the GFP substrate, the opposite is observed. This suggests the existence of regulatory factors that govern the activity of host-encoded DUBs, such as protein–protein interactions and post-translational modifications that combine to ensure the controlled removal of ubiquitin from the intended endogenous substrates. While the stable reporter cell lines described here show promise as tools for discovering DUB inhibitors, implementing orthogonal genetic and chemical screens may uncover endogenous regulators of DUB activity. Additionally, expanding the repertoire of DUB reporter cell lines might enable pooled multiplex testing of inhibitor specificity using a deep sequencing-based readout on FACS-sorted cell populations (*e.g*., GFP high vs. low). This approach would likely offer higher throughput and sensitivity compared to current multiplexed mass spectrometry strategies that use DUB activity-based probes (DUB-ABPs). In conclusion, the establishment of the reporter systems described here complements existing biochemical tools developed for studying DUBs and has the potential to both contribute to the development of inhibitors as well as uncover fundamental insights into the biology of this exciting class of enzymes.

## Materials and Methods

### Plasmids

Plasmid DNA constructs were generated by traditional molecular cloning using restriction enzyme digestion and ligation by T4 DNA ligase (New England BioLabs, #M0202L) unless stated otherwise. The UbR-EGFP fusion was generated by overlap extension PCR with deletion of the EGFP initiator methionine to prevent alternative translation initiation. The UbR-EGFP cDNA was then cloned into an MIGR1-derived simple retroviral vector which encodes a hygromycin resistance gene downstream of an internal ribosomal entry site (IRES).^68^ The cDNA sequence for SARS2 PL^pro^ was derived from pDONR207 SARS-CoV2 TEV-NSP3, a gift from Fritz Roth (Addgene plasmid #154402, GenBank # 43740578). The cDNAs encoding CCHFV^vOTU^ (GenBank #URA30283.1), YEZV^vOTU^ (GenBank #YP_010840880.1), USP7^CD-Ubl45^ (GenBank #7874; based on previous construct,^54^ and USP28^CD^ (GenBank #57646; based on previous construct^58^ were synthesized as codon-optimized gBlocks from IDT. For generating the plasmids used in transient transfection experiments, the coding sequences for all DUBs were cloned into a pcDNA5/TO backbone (ThermoFisher # V103320) fused to mCherry (C-terminal for SARS2 PL^pro^, CCHFV^vOTU^, and YEZV^vOTU^; N-terminal for USP7 and USP28). The sequence encoding the vhhGFP4 nanobody (GFPNb) was synthesized as a gBlock from IDT based on available sequence (Addgene #35575) and cloned upstream of mCherry, mCherry-USP7 and mCherry-USP28 constructs in the pcDNA5/TO backbone. To create the plasmids used to generate the USP7 and USP28 reporter cell lines by retroviral transduction, the GFPNb-mCherry-USP7/28 fusion constructs were subcloned from the pcDNA5/TO backbone into an MIGR1-derived simple retroviral vector containing a puromycin resistance gene downstream of the IRES sequence. All amino acid substitutions were generated by site directed mutagenesis using Phusion DNA polymerase (ThermoFisher #M0530L). All plasmids were verified by DNA sequencing and the specific primers used in these constructions are available upon request.

### Cell culture, transfections, and transductions

293T cells and all derived engineered lines were cultivated at 37 °C and 5% CO_2_ in DMEM (Gibco catalog number 11875093) supplemented with 10% fetal bovine serum (ThermoFisher #11965084) and penicillin–streptomycin (Gibco #15140122). All transfection reactions were done using the TransIT-LT1 transfection reagent (Mirus #MIR 2300).

To generate the 293T-UbR-C3 and GFPNb-USP7/USP28 stable cell lines, producer 293T cells were seeded in 6-well plates at a density of 3 × 10^5^ cells per well and transfected with 1 μg of plasmid retroviral transfer vector, 0.7 μg of Gag/Gag-Pol and 0.3 μg of VSV-G. 48 h post transfection, supernatants were harvested and filtered through a 0.45 μm syringe filtration unit and 200 μL was used to transduce target cells in a 6-well plate at a density of 1 × 10^5^ cells per well. 48 h post-transduction, cells were trypsinized, resuspended, and plated into a 10 cm dish in the presence of antibiotic for 5 days (150 μg/mL hygromycin or 1 μg/mL puromycin).

To generate the clonal 293T-UbR-C3 line, 293T cells were transduced with UbR-EGFP construct, selected with hygromycin, subcloned by limiting dilution, and then individual clones were screened for a low background fluorescence level and a high induction of EGFP following treatment with the proteasome inhibitor MG132.

### Flow cytometry analysis

For the analysis of DUB activity of transiently transfected cells using flow cytometry, 293T-UbR-C3 cells were seeded in 24-well plates at a density of 7.5 × 10^4^ cells per well and transfected the following day with 400 ng of the appropriate DUB-encoding plasmid. 24 h post-transfection, cells were trypsinized and analysed by flow cytometry using a BD FACSCanto II (BD Biosciences) in high-throughput 96-well plate format. FCS files were exported and data analysis was performed on gated live mCherry^+^ cells (gating strategy in Figure S1(C) using the CytoExploreR package in R studio (https://dillonhammill.github.io/CytoExploreR/).

Briefly, single-cell GFP and mCherry values were extracted across flow cytometry datasets, cells were divided into logarithmic bins based on their mCherry values, and the mean fluorescence intensities (MFI) of mCherry and GFP were calculated for each bin. These MFI values were plotted against each other, fitted with a 4-parameter logistical curve in GraphPad Prism 9, and the resulting curves were interpolated to calculate the level of DUB expression (mCherry) required to elevate GFP above a threshold determined based on the negative, non-transfected control. With the voltage settings used on our flow cytometer, this cutoff of GFP+ was set to an MFI of 1000, but this value can vary depending on cytometer voltage settings and should be determined individually using an appropriate negative control. The relative activity of WT and mutant versions of DUBs were obtained by calculating the fold difference between their respective mCherry values required to reach the threshold of GFP stabilization.

### Small molecule inhibition assays

All small molecule inhibitors were purchased from MedChemExpress, apart from XL177A, which was provided by the Buhrlage lab. For inhibition assays of SARS2-PL^pro^ using GRL0617, 293T-UbR-C3 cells were seeded at a density of 7.5 × 10^5^ cells per well in a 24-well plate and transfected the following day with 400 ng of the appropriate SARS2-PL^pro^ plasmids. Cell media was changed 6 h post transfection and replaced with drug-containing media at the appropriate concentration. 24 h post-transfection, cells were trypsinized and analyzed by flow cytometry as described above. The DUB activity values obtained at each drug concentration relative to the untreated control for each respective mutant were plotted using GraphPad Prism 9 and fit to a 4-parameter logistic curve to determine IC_50_.

For inhibition assays performed using the GFPNb^R35Q^-USP7/USP28 stable cell lines, cells were seeded at a density of 2 × 10^5^ cells per well in a 96-well plate, treated with the appropriate concentrations of inhibitor 8 h post-seeding, and analyzed by flow cytometry the following day. Given the highly uniform levels of DUB expression in the stable cell lines, drug-mediated inhibition could be quantified by directly assessing the GFP MFI of the live cell population. The GFP MFI of the inhibitor treated cells relative to the untreated controls were used to calculate relative activity and then plotted in GraphPad Prism 0 and fit to a 4-parameter logistic curve to determine IC_50_.

### Immunoblotting

Immunoblots were performed with cells harvested 24 h post transfection or 16 h post inhibitor treatment from 24 well plates that were initially seeded at a concentration of 7.5×10^5^ cells per well. Harvested cells were pelleted and resuspended in 25 μL of PBS and 25 μL of reducing sample buffer (125 mM Tris-HCl, pH 6.8, 20% glycerol, 7.5% SDS, 5% 2-mercaptoethanol, 250 mM dithiothreitol [DTT], and 0.05% bromophenol blue) and denatured at 98 °C for 15 minutes. Samples were run on an SDS-PAGE gel (4–20% Criterion TGX Precast Midi Protein Gel, Bio Rad # 5671094) and transferred to a polyvinylidene difluoride (PVDF) membrane (Millipore #IPVH00010). Immunoblots were probed with mouse anti-GFP antibody (1:5000, Clontech #632375), rabbit anti-mCherry (1:1000, Invitrogen), rabbit anti-Actin (1:10000, Cell Signaling Technology #4970) and mouse anti-Tubulin (1:10000, ThermoFisher # A11126) primary antibodies followed by goat/sheep anti-mouse IgG IRDye 680 (1:10,000; LI-COR; catalog no. 926–68070) or goat anti-rabbit IgG-horseradish peroxidase (HRP; 1:10,000; Jackson Laboratory; catalog no. 111–035–144). Membranes probed with HRP secondary, visualized using the SuperSignal West Femto maximum sensitivity substrate (ThermoFisher; catalog no. PI34095). Images were acquired using the LI-COR Odyssey Fc imaging system.

### Fluorescence microscopy

In Figures 1B and 3A, approximately 50,000 293T-Ub-R-C3 were seeded into 24-well imaging plates (Ibidi #82426) and transfected the next day with 400 ng of the indicated plasmids using TransIT-LT1 (Mirus #2304). Representative images were acquired 24 h after transfection using a Cytation 5 Cell Imaging Multi-Mode Reader (BioTek) at × 10 magnification.

### Protein structure modeling

A structural model for YEZV^vOTU^ was generated with RoseTTAFold^47^ using the ROBETTA web server and top-ranked models underwent simple all-atom refinement using the Relax application on the ROSIE 2 server.^69^ The approximate interacting surface between YEZV^vOTU^ and ubiquitin was estimated by aligning the RoseTTAFold-generated model of YEZV^vOTU^ to the co-crystal structure of CCHFV^vOTU^ covalently bound to ubiquitin (PDB: 3PHX) using PyMOL.^43^ All structural figures were generated using PyMOL.

### Phylogenetic tree generation

The amino acid sequences of the L-protein/RNA-dependent RNA polymerase from the following nairoviruses were obtained from the NCBI Protein RefSeq database: AHV (AMT75434.1), ARTSV (AKC89352.1), AVAV (AMT75377.1), BURV (AKC89349.1), CASV (AKC89346.1), CHIMV (AKC89343.1), CCHFV (AAQ98866.2), DGKV (AMT75389.1), DUGV (AAB18834.1), ERVEV (AFH89032.1), FARV (AMT75398.1), GANV (AMT75401.1), GERV (AKC89340.1), GOSV (ALD83626.1), GRSV (AMT75404.1), HAZV (AAZ38668.1), HpTV-1 (YP_009293587.1), HUGV (AKC89316.1), ISKV (AII79373.1), KTRV (YP_009361838.1), KUPEV (ABY82502.1), LPHV (BAP90971.1), NSDV (ACH99799.1), PRMV (AKC89337.1), PSV (AMT75410.1), QYBV (AKC89319.1), RAZAV (AMT75416.1), SAPV (AMT75422.1), SOLV (AMT75425.1), TcTV-1 (YP_009304986.1), TAGV (AMT75428.1), TDYV (AKC89328.1), TFAV (ALD84355.1), TILLV (AMT75431.1), TFLV (YP_009227122.1), UZAV (AKC89313.1), WzTV (YP_009304993.1), YEZV (BCU08948.1), YOGV (YP_009246486.1), ZIRV (AMT75437.1). To generate the phylogenetic tree in Figure S3, protein sequences were aligned using MUSCLE^70^ and maximum likelihood phylogenies were inferred using PhyML 3.0^71^ using the Smart Model Selection (SMS) tool^72^ and 100 bootstraps.

## Supplementary Material

1

## Data Availability

Data will be made available on request.

## Appendix A. Supplementary data

Supplementary data to this article can be found online at https://doi.org/10.1016/j.jmb.2023.168316.

## Abbreviations:

ABPs: activity based probes
DUB: deubiquitinase
EGFP: enhanced green fluorescent protein
GFP: green fluorescent protein
Nb: nanobody
Ub: ubiquitin
PL^pro^: papain-like protease
vOTU: viral ovarian tumor domain protease
BL2: blocking loop 2
SARS-CoV-2/SARS2: Severe acute respiratory syndrome coronavirus 2
USP: ubiquitin specific protease
CCHFV: Crimean-Congo hemorrhagic fever virus
YEZV: Yezo virus
WT: Wildtype
