# Supplementary material for: Cellular Assays for Dynamic Quantification of Deubiquitinase Activity and Inhibition
Source: J Mol Biol. Author manuscript; Available in PMC 2026 Aug 22. (PMC13499021; doi:10.1016/j.jmb.2023.168316)
Supplement: Supplementary file 1 [file NIHMS1949624-supplement-1.pdf]

Cellular assays for dynamic quantification of deubiquitinase activity and inhibition

Seyed Arad Moghadasi[1]*, Sofia N. Moraes[1]*, & Reuben S. Harris[2,3,†]

[1] Department of Biochemistry, Molecular Biology, and Biophysics, University of Minnesota – Twin Cities, Minneapolis, Minnesota, USA, 55455

[2] Department of Biochemistry and Structural Biology, University of Texas Health San Antonio; San Antonio, Texas, USA, 78229

[3] Howard Hughes Medical Institute, University of Texas Health San Antonio; San Antonio, Texas, USA, 78229

* These authors contributed equally to this work

[†] Correspondence: rsh@uthscsa.edu

Running title: DUB quantification in living cells

Supplement Information: Supplementary Figs. S1-S5

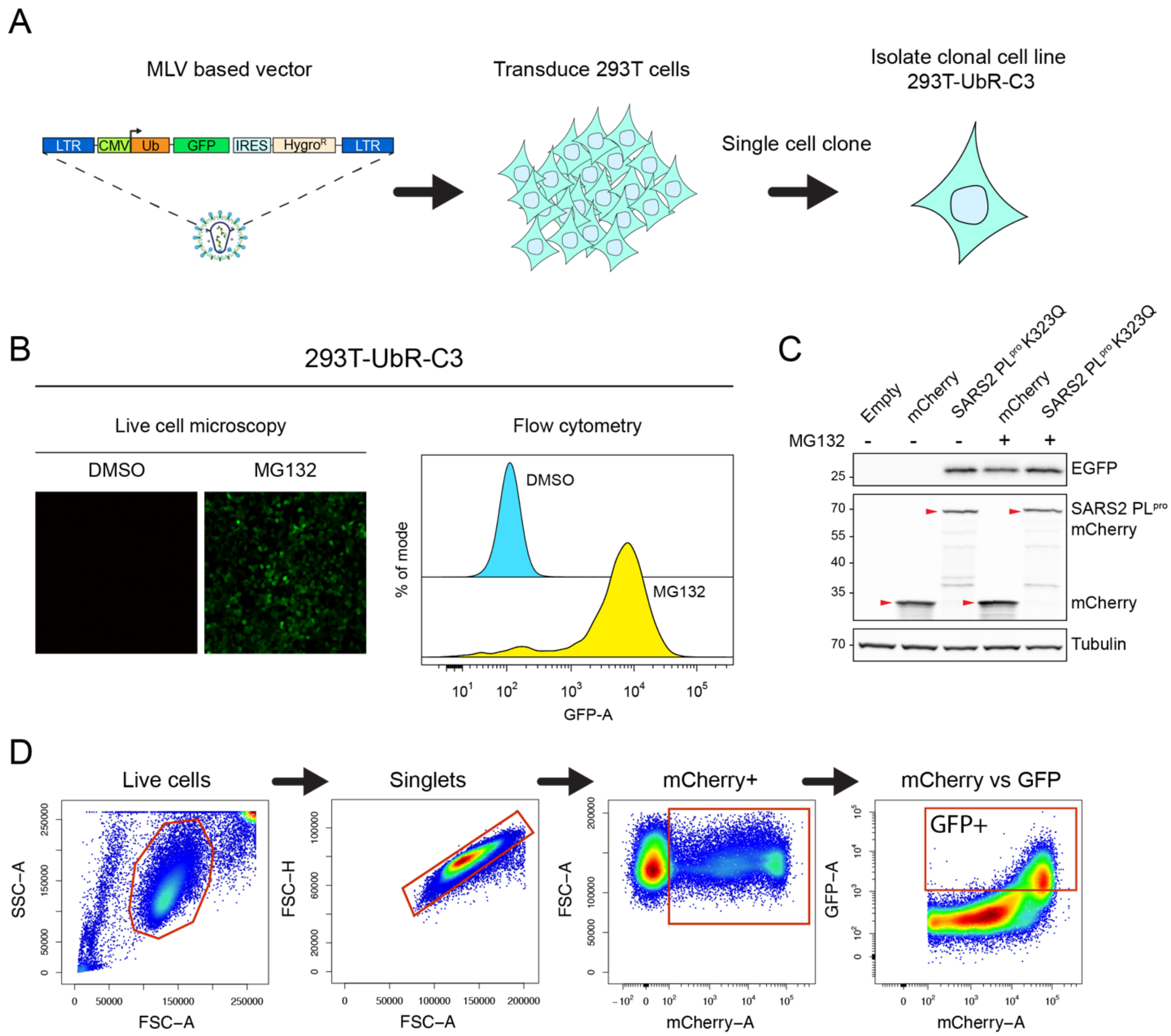

A
MLV based vector
LTR
CMV
Ub
GFP
IRES
Hygro R
LTR
Transduce 293T cells
Single cell clone
Isolate clonal cell line 293T-UbR-C3
B
293T-UbR-C3
Live cell microscopy
DMSO
MG132
Flow cytometry
DMSO
MG132
% of mode
GFP-A
C
Empty
mCherry
SARS2 PLpro K323Q
mCherry
SARS2 PLpro K323Q
MG132
EGFP
SARS2 PLpro mCherry
mCherry
Tubulin
D
Live cells
SSC-A
FSC-A
Singlets
FSC-H
FSC-A
mCherry+
FSC-A
mCherry-A
mCherry vs GFP
GFP+
GFP-A
mCherry-A

Supplementary Figure 1. **Generation and validation of a stable cell line expressing constitutively degraded EGFP.**

(**A**) Workflow of generating stable clonal cell line with a schematic of the murine leukemia virus-based vector encoding the ubiquitin-R-EGFP fusion construct used to transduce 293T cells. Transduced cells were selected with hygromycin and single cell cloned by limiting dilution to establish a maximally responsive cell line named 293T-UbR-C3.

(**B**) Representative fluorescent microscopy imaging and GFP fluorescence intensity by flow cytometry of 293T-UbR-C3 cells treated with 10μM of the proteasome inhibitor MG132 for 16h.

(**C**) Immunoblot of 293T-UbR-C3 cells transfected with mCherry or SARS2 PL^{pro} K232Q-mCherry with or without 10μM MG132 treatment (indicated by "+" or "-" symbols, respectively).

(**D**) Representative gating strategy for 293T-UbR-C3 cells transfected with SARS2 PL^{pro}-mCherry. The mCherry channel represents SARS2 PL^{pro} expression levels while the GFP channel is a proxy for DUB activity.

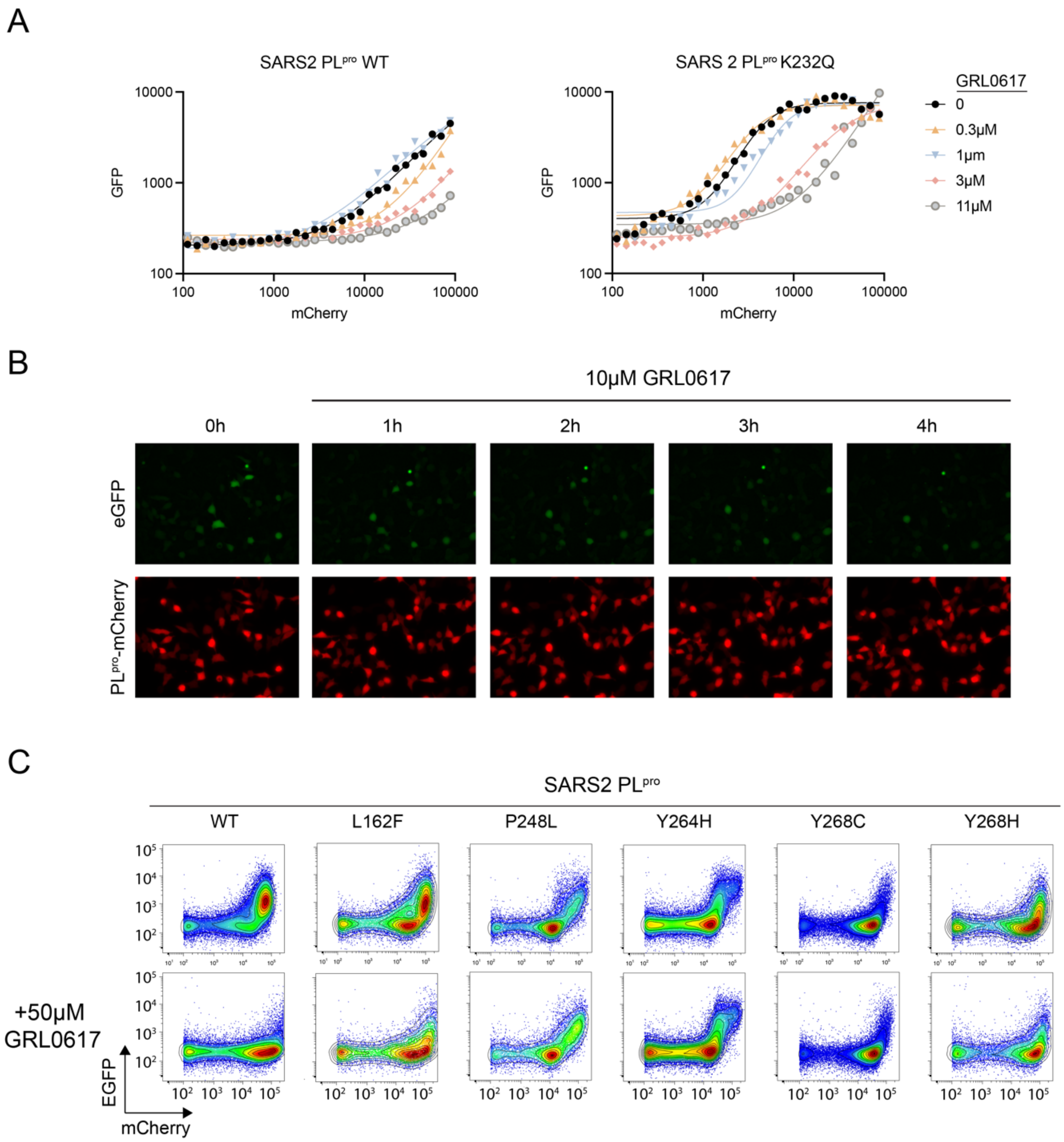
A
SARS2 PLpro WT
SARS 2 PLpro K232Q
GFP
mCherry
GRL0617
0
0.3µM
1µm
3µM
11µM
B
10µM GRL0617
0h
1h
2h
3h
4h
eGFP
PLpro-mCherry
C
SARS2 PLpro
WT
L162F
P248L
Y264H
Y268C
Y268H
+50µM GRL0617
EGFP
mCherry

Supplementary Figure 2. **Flow cytometry quantification of SARS2 PL^{pro} mutant inhibition by GRL0617.**

(**A**) Representative binned quantification of flow cytometry (analogous to Figure 1D) data for SARS2 PL^{pro}-WT and K232Q in response to the indicated concentration of GRL0617.

(**B**) Representative time-lapse fluorescence microscopy images of 293T-UbR-C3 cells transfected with SARS2-PL^{pro}-mCherry and treated with 10 μM GRL0617 at 24h post-transfection, followed by imaging at the indicated timepoints.

(**C**) Representative flow cytometry plots of cells 293T-UbR-C3 cells transfected with the indicated SARS2 PL^{pro} mutants with and without 50μM treatment of GRL0617.

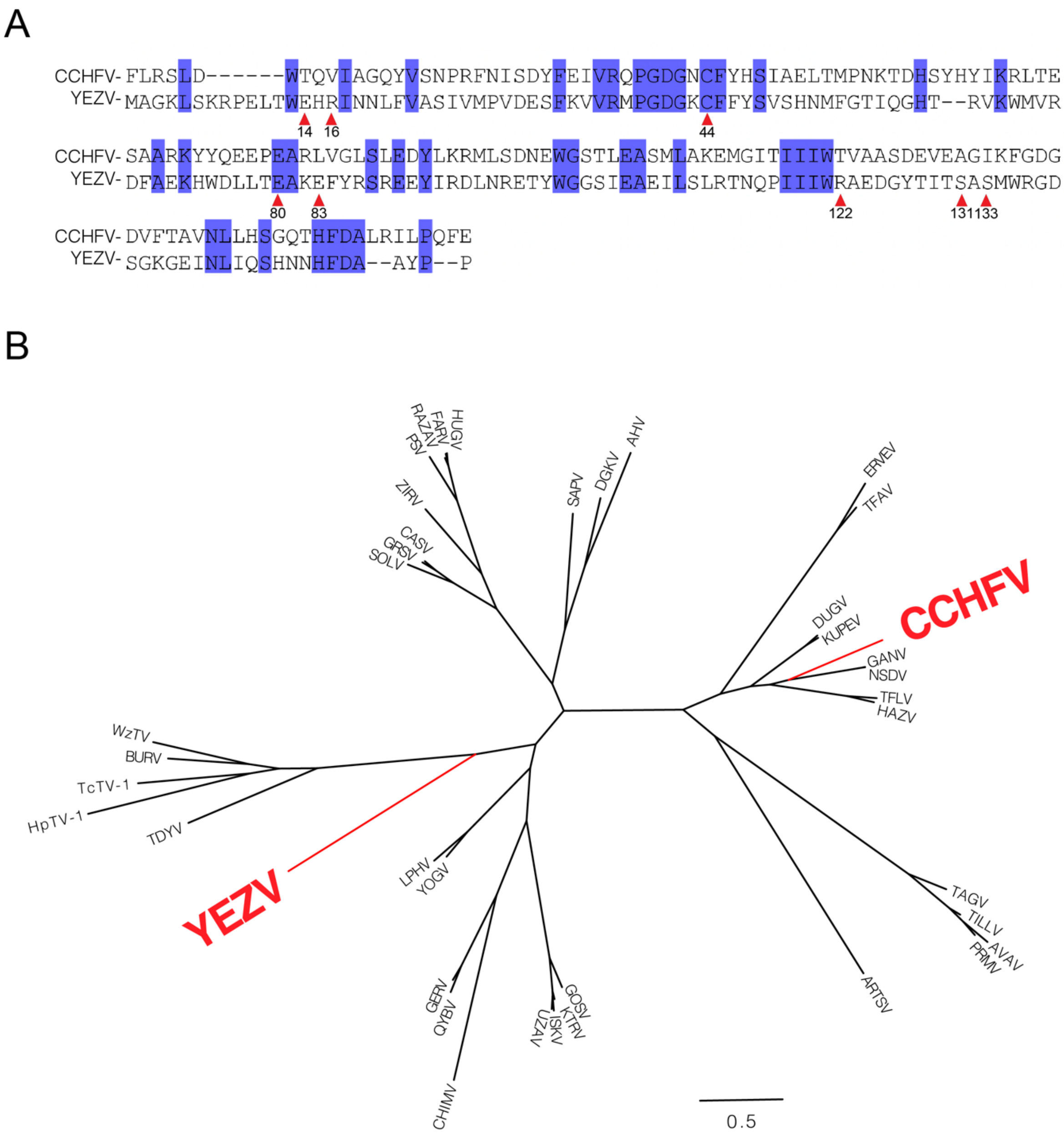
A
CCHFV- FLRSLD------WTQVIAGQYVSNPRFNISDYFEIVRQPGDGNCFYHSIAELTMPNKTDHSYHYIKRLTE
YEZV- MAGKLSKRPELTWEHRINNLFVASIVMPVDESFKVVRMPGDGKCFFYSVSHNMFGTIQGHT--RVKWMVR
14
16
44
CCHFV- SAARKYYQEEPEARLVGLSLEDYLKRMLSDNEWGSTLEASMLAKEMGITIIIWTVAASDEVEAGIKFGDG
YEZV- DFAEKHWDLLTEAKEFYRSREEYIRDLNRETYWGGSIEAEILSLRTNQPIIIWRAEDGYTITSASMWRGD
80
83
122
131
133
CCHFV- DVFTAVNLLHSGQTHFDALRILPQFE
YEZV- SGKGEINLIQSHNNHFDA--AYP--P
B
HUGV
FARV
RAZAV
PSV
ZIRV
CASV
GRSV
SOLV
SAPV
DGKV
AHV
ERVEV
TFAV
DUGV
KUPEV
CCHFV
GANV
NSDV
TFLV
HAZV
WzTV
BURV
TcTV-1
HpTV-1
TDYV
YEZV
LPHV
YOGV
GERV
QYBV
CHIMV
GOSV
KTRV
ISKV
UZAV
TAGV
TILLV
AVAV
PRMV
ARTSV
0.5

Supplementary Figure 3. **Evolutionary comparison of CCHFV and YEZV vOTU domains.**

(**A**) Amino acid alignment of vOTU domains from CCHFV and YEZV. Red arrows denote residues that were mutated in this study and the number below the arrows correspond to the amino acid position in YEZV vOTU.

(**B**) Phylogenetic tree of vOTU domains from nairoviruses. Scale bar represents evolutionary distance.

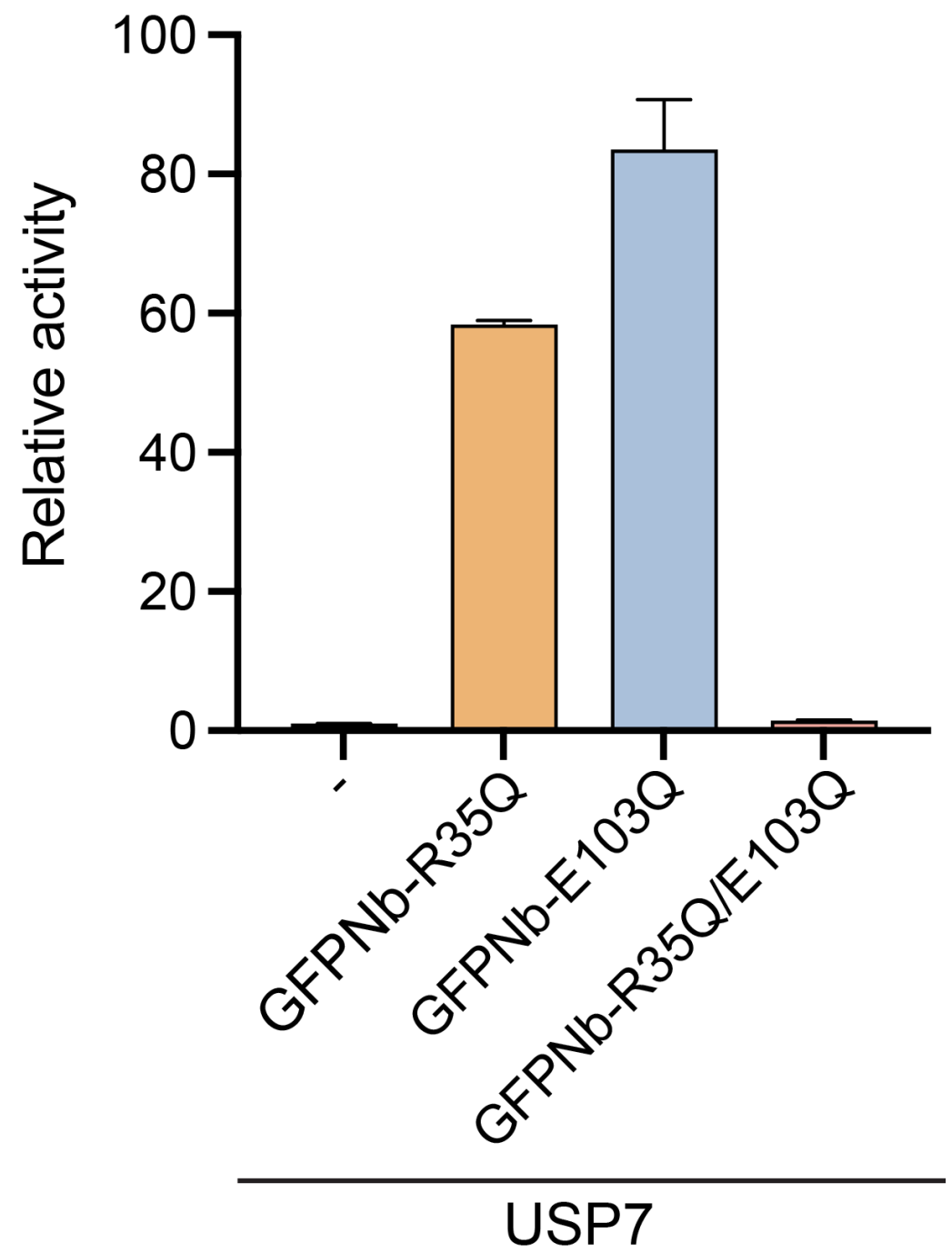
100
80
60
40
20
0
Relative activity
-
GFPNb-R35Q
GFPNb-E103Q
GFPNb-R35Q/E103Q
USP7

Supplementary Figure 4. The R35Q/E103Q double mutant nanobody abolishes USP7 recruitment to EGFP.

Relative activity of USP7-GFP nanobody mutant fusions in comparison to untagged USP7, which is normalized to 1. Data are mean ± standard deviation from two independent experiments.

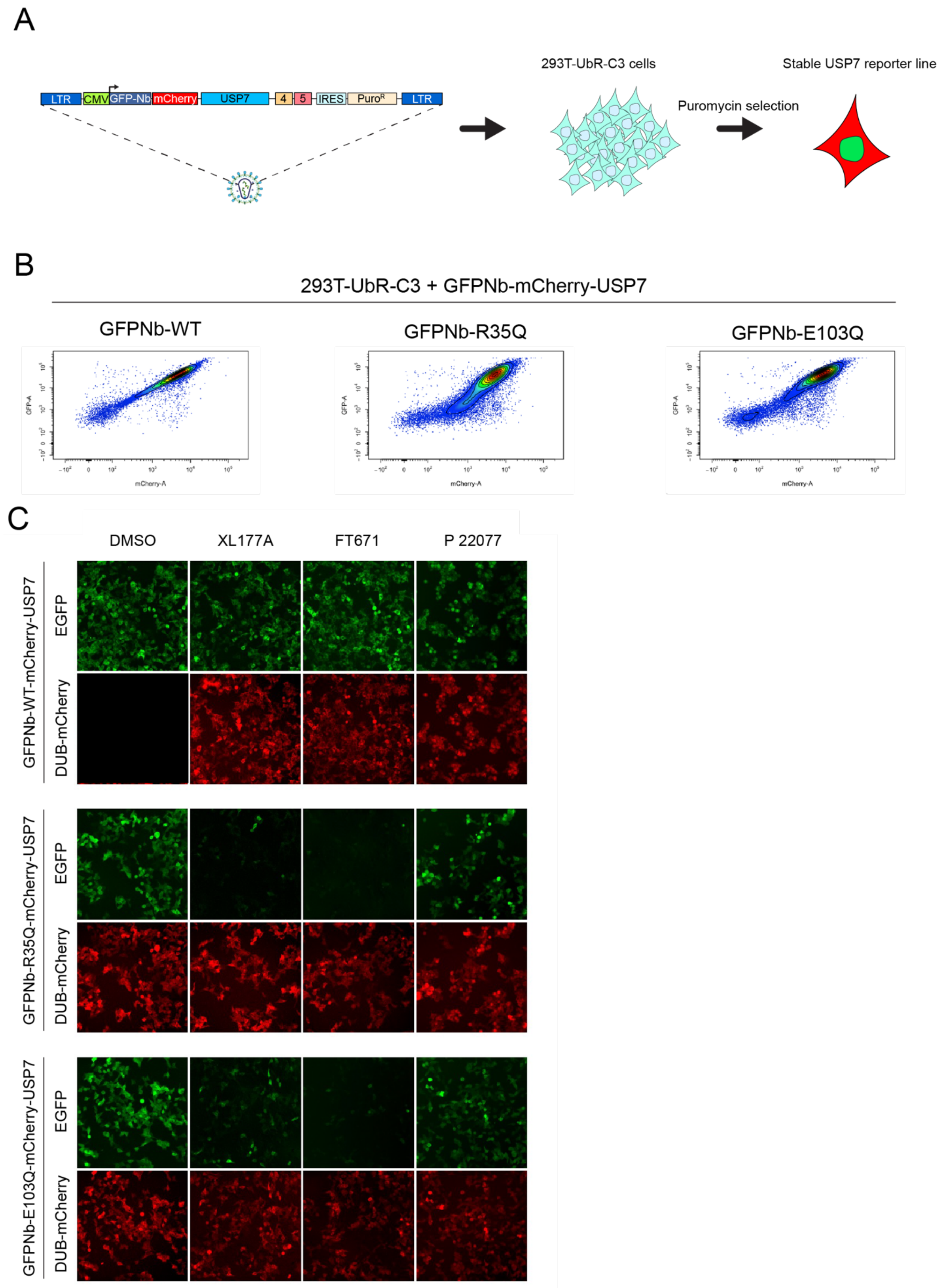

A
LTR
CMV
GFP-Nb
mCherry
USP7
4
5
IRES
Puro^R
LTR
293T-UbR-C3 cells
Puromycin selection
Stable USP7 reporter line
B
293T-UbR-C3 + GFPNb-mCherry-USP7
GFPNb-WT
GFPNb-R35Q
GFPNb-E103Q
GFP-A
mCherry-A
C
DMSO
XL177A
FT671
P 22077
GFPNb-WT-mCherry-USP7
GFPNb-R35Q-mCherry-USP7
GFPNb-E103Q-mCherry-USP7
EGFP
DUB-mCherry

Supplementary Figure 5. **Generation of stable cell lines expressing GFPNb-USP7 fusions.**

(**A**) Workflow for establishing stable cell lines. A schematic depicting a viral construct encoding the GFPNb-mCherry-USP7 fusion and a downstream IRES-puromycin resistance cassette used to transduce 293T-UbR-C3 cells. Cells were selected using 1μg/mL of puromycin to establish a pool of cells stably expressing the GFPNb-mCherry-USP7 fusions.

(**B**) Representative flow cytometry plots of a pool of 293T-UbR-C3 cells stably expressing the indicated GFPNb-mCherry-USP7 fusions.

(**C**) Representative fluorescent microscopy images of the indicated cell lines treated with 10μM XL177A, FT671 or P 22077. The GFP channel of the GFPNbWT and GFPNbR35Q fusions are identical to those in Figure 5B and shown here for comparison to the mCherry channel to demonstrate GFPNb-mCherry-USP7 expression.
